# Origin, spread, and interspecies transmission of a dominant genotype of BJ/94 lineage H9N2 avian influenza viruses with increased threat

**DOI:** 10.1093/ve/veae106

**Published:** 2024-12-09

**Authors:** Yong Zhou, Yudong Li, Hongzhuang Chen, Sicheng Shu, Zhixin Li, Honglei Sun, Yipeng Sun, Jinhua Liu, Lu Lu, Juan Pu

**Affiliations:** National Key Laboratory of Veterinary Public Health and Safety, Key Laboratory for Prevention and Control of Avian Influenza and Other Major Poultry Diseases, Ministry of Agriculture and Rural Affairs, College of Veterinary Medicine, China Agricultural University, 2 Yuanmingyuan West Road, Haidian District, Beijing 100193, China; National Key Laboratory of Veterinary Public Health and Safety, Key Laboratory for Prevention and Control of Avian Influenza and Other Major Poultry Diseases, Ministry of Agriculture and Rural Affairs, College of Veterinary Medicine, China Agricultural University, 2 Yuanmingyuan West Road, Haidian District, Beijing 100193, China; National Key Laboratory of Veterinary Public Health and Safety, Key Laboratory for Prevention and Control of Avian Influenza and Other Major Poultry Diseases, Ministry of Agriculture and Rural Affairs, College of Veterinary Medicine, China Agricultural University, 2 Yuanmingyuan West Road, Haidian District, Beijing 100193, China; National Key Laboratory of Veterinary Public Health and Safety, Key Laboratory for Prevention and Control of Avian Influenza and Other Major Poultry Diseases, Ministry of Agriculture and Rural Affairs, College of Veterinary Medicine, China Agricultural University, 2 Yuanmingyuan West Road, Haidian District, Beijing 100193, China; Ningxia Hui Autonomous Region Animal Disease Prevention and Control Center, No. 411, Mancheng South Street, Jinfeng District, Yinchuan City, Ningxia Hui Autonomous Region, Yinchuan 750011, China; National Key Laboratory of Veterinary Public Health and Safety, Key Laboratory for Prevention and Control of Avian Influenza and Other Major Poultry Diseases, Ministry of Agriculture and Rural Affairs, College of Veterinary Medicine, China Agricultural University, 2 Yuanmingyuan West Road, Haidian District, Beijing 100193, China; National Key Laboratory of Veterinary Public Health and Safety, Key Laboratory for Prevention and Control of Avian Influenza and Other Major Poultry Diseases, Ministry of Agriculture and Rural Affairs, College of Veterinary Medicine, China Agricultural University, 2 Yuanmingyuan West Road, Haidian District, Beijing 100193, China; National Key Laboratory of Veterinary Public Health and Safety, Key Laboratory for Prevention and Control of Avian Influenza and Other Major Poultry Diseases, Ministry of Agriculture and Rural Affairs, College of Veterinary Medicine, China Agricultural University, 2 Yuanmingyuan West Road, Haidian District, Beijing 100193, China; Roslin Institute, University of Edinburgh, Easter Bush Campus, Midlothian, Edinburgh EH2 59RG, United Kingdom; National Key Laboratory of Veterinary Public Health and Safety, Key Laboratory for Prevention and Control of Avian Influenza and Other Major Poultry Diseases, Ministry of Agriculture and Rural Affairs, College of Veterinary Medicine, China Agricultural University, 2 Yuanmingyuan West Road, Haidian District, Beijing 100193, China

**Keywords:** H9N2 avian influenza viruses, origin, transmission pattern, reassortment, public health

## Abstract

The H9N2 subtype of avian influenza viruses (AIVs) is widely prevalent in poultry and wild birds globally, with occasional transmission to humans. In comparison to other H9N2 lineages, the BJ/94 lineage has raised more public health concerns; however, its evolutionary dynamics and transmission patterns remain poorly understood. In this study, we demonstrate that over three decades (1994–2023), BJ/94 lineage has undergone substantial expansion in its geographical distribution, interspecies transmission, and viral reassortment with other AIV subtypes, increasing associated public health risks. These changes were primarily driven by the emergence of a dominant genotype G57. In the first decade, G57 emerged in East China and rapidly adapted to chickens and spread across China. Since 2013, the G57 genotype has expanded beyond China into eight other countries and reassorted with various AIV subtypes to form new zoonotic reassortants. Chickens have played a key role in the generation and circulation of the G57 viruses, with ducks and other poultry species likely assuming an increasingly importantly role. Over the past decade, G57 has been more frequently detected in wild birds, mammals, and humans. Additionally, Vietnam has emerged as a new hotspot for the international spread of G57. Our results suggest that the BJ/94 lineage H9N2 virus may continue to overcome geographical and species barriers, with potentially more severe consequences.

## Introduction

The H9N2 avian influenza virus (AIV) is a significant global concern, threatening the sustainable development of the agricultural industry while continuing to challenge public health and safety (Peacock et al. 2019, Carnaccini and Perez 2020, Bi et al. 2022). H9N2 viruses have been classified into two main lineages according to the phylogeny of HA gene sequences, the American lineage and the Eurasian lineage (Carnaccini and Perez 2020). The Eurasian lineage can be further subdivided into three sublineages: BJ/94 (or Y280), G1, and Y439 (Guan et al. 1999). The BJ/94 lineage H9N2 viruses are predominantly endemic to poultry in China (Carnaccini and Perez 2020). Nearly 80% of all publicly available H9N2 HA sequences belong to the BJ/94 lineage. The G1 lineage H9N2 viruses are mainly prevalent in poultry in the Middle East (Arai et al. 2019). Meanwhile, the Y439 lineage H9N2 viruses are endemic in wildfowl and have spread globally, but a distinct Y439 subcluster has evolved in poultry in Korea (Peacock et al. 2019, Carnaccini and Perez 2020). H9N2 viruses belonging to the American lineage are prevalent among wild birds, especially seabirds, in the Americas (Peacock et al. 2019).

China is widely recognized as one of the regions most severely affected by BJ/94 lineage H9N2 virus. Since the first isolation of BJ/94 lineage H9N2 virus in China in 1994 (Li et al. 2005), the virus has continued to circulate for three decades. Currently, the H9N2 virus holds the highest isolation rate among AIV subtypes that have been identified in poultry farms (Bi et al. 2020) and is known to reduce egg production in laying hens and impair broiler performance (Guo et al. 2000, Bonfante et al. 2018, Jonas et al. 2018).

BJ/94 lineage H9N2 viruses have undergone significant evolutionary changes. Prior to 2010, the epidemic of H9N2 viruses in China was characterized by a high level of diversity, with multiple genotypes cocirculating (Sun et al. 2010, Wei et al. 2016, Li et al. 2017). However, a new genotype of G57 emerged in the BJ/94 lineage H9N2 viruses in 2007, the virus rapidly spread across China after 2010, replacing other H9N2 genotypes. This shift marked the first observed “genetic bottleneck” within the BJ/94 lineage (Pu et al. 2015). Meanwhile, there has been a dramatic increase in human cases of BJ/94 lineage infections (Adlhoch et al. 2023). Notably, the BJ/94 lineage H9N2 viruses contributed all six internal genes to various novel AIVs capable of cross-species transmission to humans, including H7N9, H10N8, H5N6, and H3N8 identified between 2013 and 2023 (Bi et al. 2016; Chen et al. 2014, Pu et al. 2015; Yang et al. 2022). However, it remains unclear whether the internal genes of these reassortants are exclusively derived from the G57 genotype.

Collectively, these evidences suggested that H9N2 AIVs, particularly those from the BJ/94 lineage, represent an increasing threat to both avian and human populations due to their continuous evolution, highlighting the urgent need for further investigation. This study comprehensively examines the genetic evolution process of BJ/94 H9N2 virus, focusing on understanding the evolution and transmission of its dominant genotype, as well as its reassortment with other AIV subtypes. Our findings provide crucial insights to improve understanding and inform more effective strategies for the prevention and control of H9N2 virus.

## Methods

### Sequence data

H9N2 genome (contains eight gene segments) sequences were obtained from the Global Initiative on Sharing All Influenza Data (GISAID) (https://platform.epicov.org/) and National Center for Biotechnology Information (NCBI) Influenza Virus Resource (https://www.ncbi.nlm.nih.gov/genomes/FLU/) databases on 30 March 2023. We then removed duplicate isolates from both databases and laboratory-derived isolates. In total, we obtained 5266 H9N2 viruses with full-length or near full-length genome sequences, and more detailed information on the viral sequences is provided in Supplementary Table S1.

### Molecular and phylogenetic analyses

We performed a multiple sequence alignment for each of the eight gene segments of H9N2 viruses using MAFFT v7.0 (Katoh and Standley 2013) and reconstructed maximum likelihood (ML) trees for eight genes separately using IQ-TREE v1.6 (Nguyen et al. 2015), performing ultrafast bootstrap resampling analysis (1000 replications), and using the best-fitted nucleotide substitution model (HA, NA, PB2, PB1, PA, NP, M, and NS were GTR + F + R10, GTR + F + R7, GTR + F + R8, GTR + F + R8, GTR + F + R7, GTR + F + R9, TVM + F + R8, and TVM + F + R7, respectively) (Kalyaanamoorthy et al. 2017). Trees were visualized and annotated by using FigTree v1.4.4 (http://tree.bio.ed.ac.uk/software/figtree/), Adobe Illustrator 2021, and iTOL (https://itol.embl.de/) (Letunic and Bork 2019).

### Homology analysis

To investigate how H9N2 genes, especially internal segments, reassort with other AIV subtypes, we integrated the genome sequence datasets of AIV from all subtypes in China, sourced from both GISAID and NCBI databases using a customized Python script. We conducted homology analysis of each segment of the H9N2 virus and other subtypes separately, using the Basic Local Alignment Search Tool (BLAST), with a sequence identity threshold set at 97%. Subsequently, Kernel Density Estimation (KDE) plots were generated using the Seaborn package in Python.

### Selection pressure analysis

The ratio of nonsynonymous to synonymous substitution rates (Ka/Ks) of HA protein was calculated using the software KaKs_Calculator 3.0 (Zhang 2022). To maintain the accuracy of the analysis, we removed the nucleotide sequences of HA gene under 1600 bp and omitted data from 1996 to 1999 due to an insufficient number of sequences. The sequences were then converted into pairwise alignments in quasi-AXT format as input, and mean Ka/Ks ratios were collected for analysis of selection pressure.

### Mutational amino acid analysis of H9N2 HA protein

To identify key mutations correlated with the distinct clades within the ML tree of the H9N2 HA gene, fixed mutations were inferred using R package sitePath (Ji et al. 2022). All the fixed mutations were detected, and the results were visualized using the R package ggtree (Xu et al. 2022).

### Phylodynamic analysis

#### Subsampling and analysis strategies for the G57 H9N2 virus gene origin datasets

According to the classification of Pu et al. (2015), we used CD-hit v4.8.1 to cluster eight gene sequences of the G57 H9N2 virus according to the 95% threshold (Fu et al. 2012) and retained only one strain in the same cluster that was isolated from the same year, same host, and same location. We used early strains to identify preisolation sequences using BLAST and added them to the subsampled dataset for analysis. We used subsampled G57 AIV datasets to conduct Bayesian phylogenetic analyses. We assembled 190, 245, 232, 216, 282, 269, 327, and 212 sequences for PB2, PB1, PA, HA, NP, NA, M, and NS genes, respectively. After three consistent subsampling repetitions, the ML phylogenetic tree was then constructed using Fasttree v2.1 (Price et al. 2010) and the temporal signals of the sequences were detected by importing the phylogenetic tree and temporal traits into TempEst (Supplementary Fig. S1) (Rambaut et al. 2016).

#### Bayesian phylogeny reconstruction

To understand the spatial dynamics of G57 H9N2 viruses, phylogeographic analyses were performed using BEAST package. We first labeled location information of all the tip sequences using two ways. First, we labeled the country trait for locations outside of China and labeled the province trait for locations within China as the vast majority of G57 H9N2 sequences were from this country. Second, we divided China into seven regions in geographic traits, namely, Central China, North China, East China, South China, Northwest China, Southwest China, and Northeast China (Supplementary Table S2. In this way, there are a total of 15 geoclusters. The host information of sequence isolates was also tested. We included the following categories: (i) major domestic birds were labeled using their common names (e.g. chicken/duck/goose), (ii) human, (iii) minor poultry composed of isolates from other domestic poultry (i.e. quail, pigeon, and ostrich), (iv) isolates from a variety of wild bird species were labeled as wild birds, and (5) nonhuman mammal species and labeled as mammals. Isolates from”environment” or unspecified host”avian” were not included in the subsampling. Time-scaled phylogenetic trees were reconstructed using BEAST v1.10.4 (Drummond et al. 2012, Suchard et al. 2018), with a General Time Reversible nucleotide substitution model with gamma distribution of substitution rates, a Gaussian Markov Random Field Skyride coalescent model, and the strict clock. Four independent Markov Chain Monte Carlo (MCMC) runs were performed for each segment, with each run consisting of 50 million iterations and samples taken every 5000 steps. A discrete trait geographic diffusion model was performed ( Trovão et al. 2015). Multiple chains were then combined after a 10% burn-in using LogCombiner v1.10 included in the BEAST package and ensure that the Effective Sample Size in Tracer v1.6 was over 200 (http://beast.bio.ed.ac.uk/Tracer). Asymmetric discrete trait model with Bayesian Stochastic Search Variable Selection (BSSVS) was performed to infer the transmission between locations and hosts. The rates of transmission and related Bayes factors (BFs) (Lemey et al. 2009) were estimated using SpreaD3 (Bielejec et al. 2016). Rates were considered statistically supported when BF > 3.0 and strongly supported when BF > 100. The maximum clade credibility (MCC) trees were summarized with a 10% burn-in removed using TreeAnnotator v1.10 in the BEAST package, and visualization was performed using FigTree v1.4.4.

We applied a discrete trait model with an additional BSSVS analysis to assess BF support and a Continuous Time Markov Chain model to estimate the transmissions between geographic states and between host states along the nodes of the trees (Minin and Suchard 2008, Su et al. 2015). The number of jumps between states was expressed as a proportion of the total number of transitions occurring across the tree (Markov jumps) and depicted as heatmaps. Heatmaps were plotted using TBtools (Chen et al. 2020). To quantify the proportion of time spent by the virus in each of these geographic states, the time spent in a particular state (Markov rewards) was also estimated (Minin and Suchard 2008). The reconstructed dispersal history in discrete space was visualized using SpreaD3 and qualitatively assessed (Bielejec et al. 2016).

Summary MCC trees for the post-burn-in posterior time-scaled trees with spatial location and host reconstructions were created using TreeAnnotator. The MCC trees contained estimates of the time, location, and host at each internal (ancestral) node. The origin of eight gene segments was estimated by finding the most recent common ancestor (MRCA) node of each gene of the G57 H9N2 virus.

## Results

### Diversification of H9N2 AIVs globally

**Figure 1.**
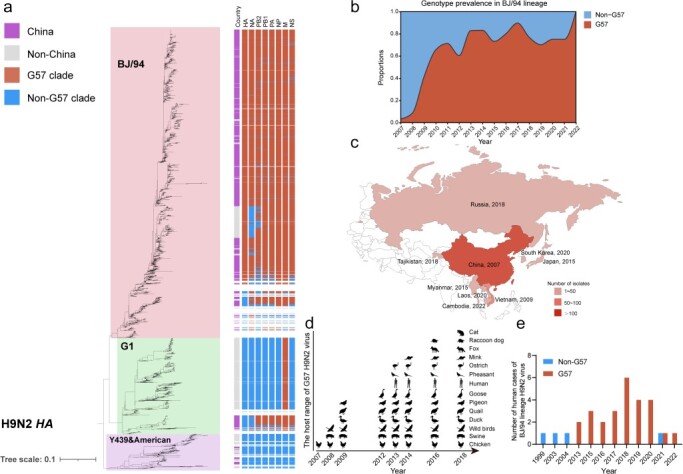
Evolution and epidemiology of the H9N2 virus. (a) Phylogenetic tree of global H9N2 virus HA genes, annotated for strains isolated after 2007. (b) Stream chart showing the proportion of G57 and non-G57 H9N2 virus isolates in BJ/94 lineage after 2007. (c) The global spread range of G57 H9N2 viruses, with the numbers representing the year when the virus was first isolated in the indicated country. The darker the color, the higher the number of isolates. (d) Host range of G57 H9N2 viruses over time. (e) Number of G57 and non-G57 H9N2 viruses isolated in humans in BJ/94 lineage (with near full-genome sequences).

We constructed phylogenetic trees for eight genes of the global H9N2 influenza viruses and identified their lineages/clades according to the previous classifications (Supplementary Fig. S2, Supplementary Table S1) (Pu et al. 2015, Carnaccini and Perez 2020). We found that BJ/94 lineage is mainly prevalent in China, and the genotype G57 identified previously according to eight gene combination remains dominant epidemic in this lineage (Fig. 1a). The temporal changes in the spatial and host distribution of G57 (Fig. 1b and Supplementary Table S3) show that in 2007, G57 only accounted for 3% of the total number of H9N2 viruses in BJ/94 lineage. However, by 2010, their prevalence exceeded 50%, and this dominant prevalence has been maintained through to 2023 (when this study dataset was curated) (Fig. 1b). Its prevalent regions have expanded from Zhejiang and Jiangxi provinces to all of China (Supplementary Fig. S3) and then to eight neighboring countries, including Vietnam, Japan, Myanmar, Russia, Tajikistan, Laos, South Korea, and Cambodia (Fig. 1c and Supplementary Table S3).

In addition, the host range of the G57 H9N2 virus has expanded to include a wide variety of poultry species, such as chickens, ducks, geese, pigeons, quail, and others, as well as in a broad range of wild birds and mammals, including swine, mink, and others (Fig. 1d and Supplementary Table S3). Notably, the number (26 cases) of human infections with G57 H9N2 viruses is significantly higher than that (4 cases) of non-G57 H9N2 viruses (Fig. 1e). This indicates that the G57 H9N2 virus remains the dominant genotype in the BJ/94 lineage and poses an escalating threat to both poultry industry and public health.

### Reassortment of BJ/94 lineage H9N2 virus with other subtype AIVs

**Figure 2.**
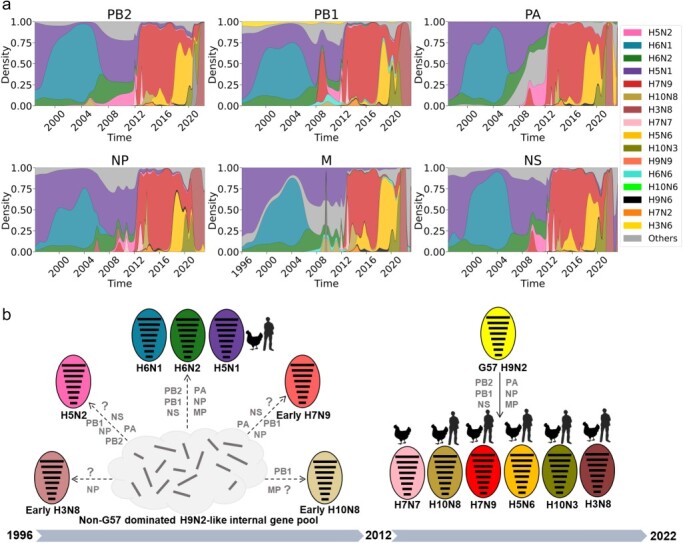
Reassortments of H9N2 viruses in China. (a) Quantitative analysis of high homology subtypes of influenza virus with H9N2-like internal segments in China. KDE with bandwidth as 0.8 year is used to calculate the relative frequency at given time points. (b) Reassortment of H9N2 with other AIV subtype viruses in China before and after 2012. Question mark indicates that the internal segment is not from the H9N2-like internal gene pool. The cartoon symbol of a person indicates that this subtype has previously infected humans, while the cartoon chicken signifies that this subtype is prevalent among chickens.

We performed homology analysis to understand how the BJ/94 lineage H9N2 virus reassorts with other AIV subtypes found in China (Fig. 2a). Our findings unveil two reassortment processes. During the period from 1996 to 2012, H5N1, H5N2, H6N1, H6N2, and other early waterfowl-derived isolates including H3N8, H7N9, and H10N8 (distinct from the isolates infecting humans since 2013) were found to be highly homologous to part or all of the H9N2-like internal genes (Fig. 2aand b), which were mainly non-G57 internal genes (Supplementary Fig. S4). Among these early reassortants, only the H5N1 virus was reported for cross-species human infections (Antigua et al. 2019). Since the dominant prevalence of G57 genotype, especially after 2012 (2013–22), a variety of new reassorting viruses have emerged including H7N9, H7N7, H10N8, H5N6, H10N3, and H3N8. These reassortants have the same reassorting pattern, i.e. they have acquired all six internal genes from the G57 genotype virus (Fig. 2a and b and Supplementary Fig. S5). Among these novel reassortants, five out of six viruses (H7N9, H10N8, H5N6, H10N3, and H3N8) caused human infections for the first time and most of them become new epidemic subtypes in chickens (Bi et al. 2016, Pu et al. 2015; Qi et al. 2014; 2022, Szablewski et al. 2023; Yang et al. 2022). Thus, the dominance of the G57 genotype after 2012 altered the reassortment pattern of BJ/94 H9N2 AIVs and most of these novel reassortants may exhibit enhanced adaptation to chicken populations and increased potential for cross-species infection.

### Selection pressure and key amino acid mutations of the H9N2 virus in China

**Figure 3.**
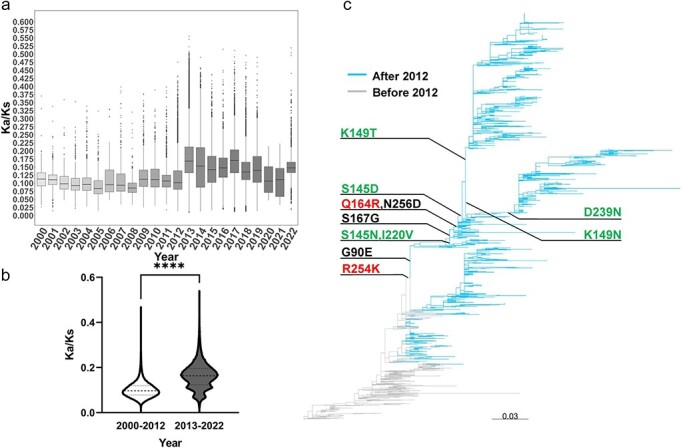
Selection pressure and key amino acid mutations of H9N2 HA protein in China. (a) Selection pressure on HA protein of the H9N2 virus from 2000 to 2022. The selection pressures of HA proteins are assessed by the Ka/Ks ratio. (b) Differential analysis of Ka/Ks ratios: comparison before and after 2012. ****P < 0.0001. (c) Amino acid mutations associated with post-2012 clades. All fixed mutations forming post-2012 clades have been annotated on the phylogenetic tree. Mutations identified as potential antigenic sites are marked in green, and those associated with mammalian adaptation are marked in red. Potential key amino acid sites that have not been reported are marked in black.

We further analyzed the selection pressure on the H9N2 virus in China and found that the HA protein of H9N2 had a shift in the selection pressure, with the timing of this shift aligning with the reassortment patterns (Figs 2b and 3a). Before 2012, the Ka/Ks ratios of HA protein are <0.125. However, since 2013, most of the Ka/Ks ratios are above apparently this value and show a significant (*P* < 0.0001) change in selection pressure (Fig. 3b). Further analysis showed that most isolates since 2013 form distinct clusters that are likely attributed to amino acid mutations with critical biological characteristics (Fig. 3c). For example, 164 and 254 positions of HA protein have been previously identified as mammalian adaptation sites (Liu et al. 2023; Zhang et al. 2023b); 145, 149, 220, and 239 positions have been previously identified as potential antigenic sites (Zhang et al. 2023a); the K149N substitution in recent H9N2 AIV is identified to contribute to viral reduced hemagglutination activity (Wen et al. 2024). These findings indicate that since 2013, the HA protein of H9N2 has been subjected to heightened selection pressure and undergone amino acid changes that might be more adaptable to poultry and mammals.

### Bayesian inference of temporal, spatial, and host origins of the G57 genotype

**Figure 4.**
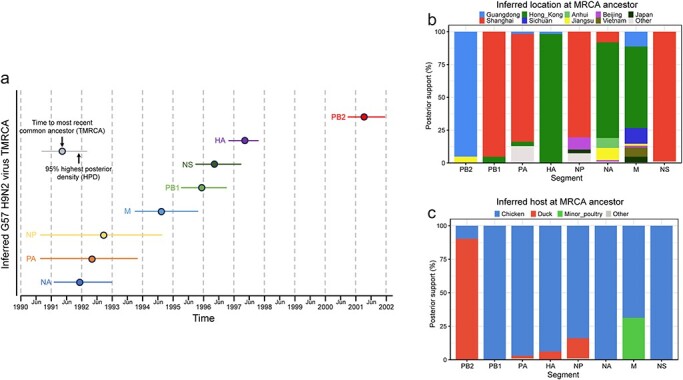
Estimated temporal, spatial, and host origins of the eight genes of the G57 H9N2 virus. (a) Inferred tMRCA of the eight genes and their 95% HPD interval. (b) Inferred origin regions and probabilities of the eight genes. (c) Inferred origin hosts and probabilities of the eight genes.

We inferred the time to most recent common ancestor (tMRCA) and determined the origins of the region and host for each of the eight genes (Supplementary Fig. S6 and Supplementary Table S4). The results showed that the NA gene of the G57 H9N2 virus had the earliest time of origin, with its tMRCA inferred to be December 1991 (95% Highest Posterior Density (HPD) from January 1991 to December 1992), while the latest was the PB2 gene, with a tMRCA of March 2001 (95% HPD from August 2000 to November 2001). tMRCAs for other genes are as follows: April 1992 for the PA gene, July 1992 for the NP gene, June 1994 for the M gene, December 1995 for the PB1 gene, May 1996 for the NS gene, and April 1997 for the HA gene (Fig. 4a and Supplementary Table S4). Additionally, according to the analysis of BSSVS, the PB1, PA, NP, and NS genes most likely originated from Shanghai in the east of China, while the HA, NA, and M genes most likely originated from Hong Kong in the south of China and the PB2 gene most likely originated from Guangdong in the south of China (Fig. 4b and Supplementary Table S4). Regarding the host origin, it was inferred that the PB1, PA, HA, NP, NA, and NS genes most likely originated from chickens, while the PB2 gene most likely originated from domestic ducks. Although the M gene was most likely to have originated from chickens, there was also a high probability that it originated from minor poultry (Fig. 4c and Supplementary Table S4).

The results highlight that the generation process of G57 spanned a minimum duration of 10 years and that chickens and ducks in East and South China contribute to the G57 H9N2 virus emergence.

### Geographic dissemination of the G57 H9N2 virus

**Figure 5.**
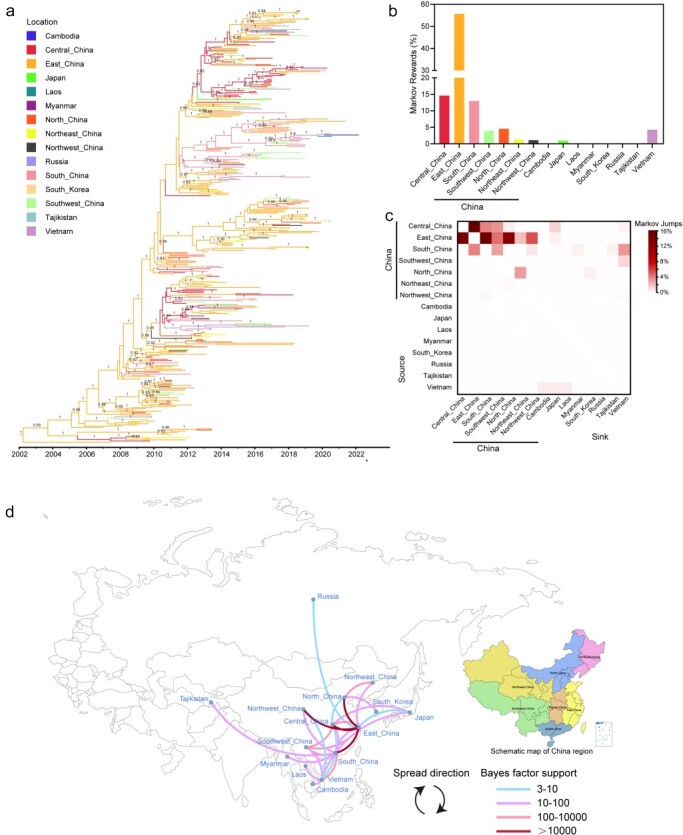
Regional spread of the G57 H9N2 virus. (a) MCC tree with branches representing different geographic regions. The posterior probabilities of host taxa over 0.8 are annotated in the nodes. (b) The proportion of time the virus spends in each location is indicated by the bar charts. (c) The frequency of transitions between locations estimated using a discrete trait phylogenetic model, and the number of location transitions was determined using Markov jumps. (d) Transitions and BFs of transmission between regions.

To explore the key regions in spread of G57 H9N2 viruses, we reconstructed the global phylogeography of G57 H9N2 viruses using a discrete trait model based on 15 geoclusters (Fig. 5a). Discrete phylogeography confirmed that East China predominantly seeded G57 H9N2 viruses, with 55.63% Markov rewards (denoting time spent in the region) and 54.79% of Markov jumps (denoting region transitions) with decisive support (BF > 100) (Fig. 5b–d and Supplementary Fig. S7A and Supplementary Table S5). Central and South China accounted for 14.59% and 12.98% of the Markov rewards and 24.83% and 11.35% of the Markov jumps, respectively (Fig. 5c and Supplementary Table S5).

International transmissions of G57 are mainly from Central China to Japan (Markov jumps 1.34%, BF = 37.48) and from South China to Vietnam (Markov jumps 3.18%, BF = 824.70) (Fig. 5c and d and Supplementary Fig. S7B and S7C and Supplementary Table S5). Notably, Vietnam plays an important role in the transition of G57 H9N2 viruses in Southeast and East Asian countries, accounting for 4.26% of Markov rewards, which was the fifth highest among all regions. The BFs for Vietnam’s transmission to Cambodia, Laos, and Japan are between 63.82 and 91.81 (Fig. 5 and Supplementary Fig. S7D and Supplementary Table S5).

Collectively, our analyses on the origin and transmission patterns of the G57 inferred that through the acquisition of genes from Hong Kong, Shanghai, and Guangdong, the G57 virus eventually emerged in East China, a process that took about 10 years from 1991 to 2002 (Fig. 5a and Supplementary Fig. S8a and Supplementary Fig. S6). Following its emergence, G57 outbreaks were initially observed only in East China between 2002 and 2005. Around 2006, the virus spread to Central China, followed by Southwest China in 2007 and South China and Vietnam by 2008. Subsequently, the G57 H9N2 virus further expanded from East and Central China to North, Northwest, and Northeast China by 2009, eventually spreading across the entire country. By 2014, the virus had to spread from Central and South China, as well as Vietnam, to Japan and Myanmar. By 2016, the virus had spread from East and South China to Russia and Tajikistan, respectively. By 2019, the virus had spread from North China to South Korea, and by 2020, the virus had spread from Vietnam to Laos. Furthermore, around 2021, the virus further jumped from Vietnam to Cambodia (Supplementary Fig. S8b–8e and Supplementary Movie 1). The findings indicate that the G57 virus took approximately a decade (2002–12) to spread from eastern China to the entire country, followed by another decade (2013–22) to reach eight additional countries. This pattern highlights the virus’s potential for further geographical expansion.

### Cross-species transmission of the G57 H9N2 virus

**Figure 6.**
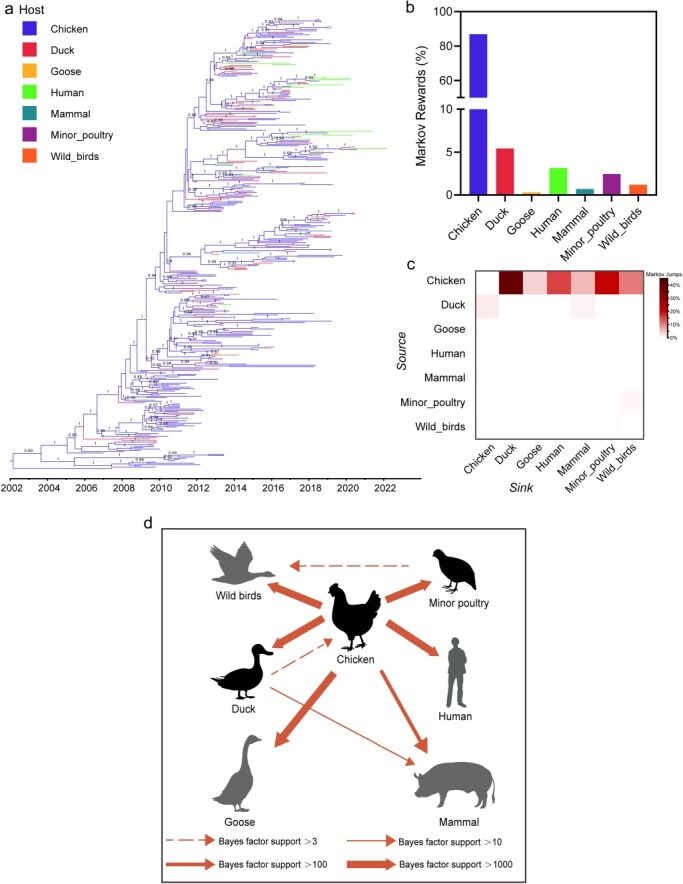
Cross-species transmission of the G57 H9N2 virus. (a) MCC tree with branches representing different hosts. The posterior probabilities of host taxa over 0.8 are annotated in the nodes. (b) The proportion of time the virus spends in each host taxa is indicated by the bar charts. (c) The frequency of transitions between host pairs was estimated using a discrete trait phylogenetic model, and the number of transitions between species/taxa was determined using Markov jumps. (d) The host interfaces between the various host taxa are inferred based on host transitions that are well supported by BF. Overspill hosts are shown in black.

We quantified the contributions of different host types in the cross-species transmissions of G57 AIVs (Fig. 6a). The results showed that chickens were the absolute major source responsible for the transmission of G57 H9N2 viruses, accounting for 86.83% of Markov rewards and 96.15% of Markov jumps (Figs 6b, 6c and Supplementary Table S6). Specifically, the transmission from chicken to duck, goose, minor poultry, wild birds, human as well as mammals was supported by strong BFs (BF > 100) (Fig. 6d and Supplementary Table S6), and their Markov jumps were 40.05%, 3.54%, 21.98%, 10.36%, 14.83%, and 5.39%, respectively (Fig. 6c and Supplementary Table S6). Additionally, ducks also played an important role in the transmission of G57 H9N2 viruses, accounting for 5.43% of Markov rewards and 2.36% of Markov jumps (Fig. 6b and c and Supplementary Table S6), and the direction of its transmission was mainly to mammals and to chicken, which was also supported by the BFs (3 < BF < 100), suggesting a bidirectional transmission between chickens and ducks (Fig. 6d and Supplementary Table S6). Besides, transmission from minor poultry to wild birds is also strongly supported (3 < BF < 100) (Fig. 6d and Supplementary Table S6). This implies that wild birds entering the transmission chain of G57 H9N2 virus may make it easier for the virus to spread over long distances.

## Discussion

The present study has identified that the increased public1 health threat posed by lineage BJ/94 is likely to be associated with the emergence and widespread transmission of the dominant G57 genotype. Furthermore, the H9N2 virus has shown the ability to spread across different regions and infect a wide range of hosts. This adaptability suggests that it could become an increasing public health concern in the future.

Previous phylogenetic studies revealed that early H9N2 viruses in mainland China may have originated from Hong Kong (Jin et al. 2014; Yang et al. 2019). Our analysis supports these findings, showing that Hong Kong was the origin of the NA, M, and HA genes of the G57 H9N2 virus, underscoring its crucial role in the genesis of the H9N2 viruses. Furthermore, our analysis identified Shanghai and Guangdong as additional sources of gene segments for the G57 H9N2 virus. We inferred the emergence of the G57 H9N2 virus in May 2002. Since then, the virus likely circulated at a low level in East China for 4–5 years. Around 2006, the epidemiological range of the virus began expanding into Central China, leading to its first isolation in 2007 in Jiangxi (Central China) and Zhejiang (East China). We acknowledge the common challenge for such analysis that there is potential bias resulting from inconsistent sequencing efforts across different times and regions. Previous studies have shown that high-prevalence areas, such as regions with widespread H9N2 infections in Chinese chicken flocks, are over-represented in sequence datasets, primarily reflecting local transmissions (Carnaccini and Perez 2020; Fusaro, 2024). To address this bias in the analysis of public sequence data, we applied a downsampling approach based on region, host, and time while preserving the original proportional distributions.

In our analysis of the geographic spread of G57 H9N2 viruses, we found that East China, Central China, South China, and Vietnam were important sources and major epidemic areas, accounting for 45.61%, 12.29%, 16.31%, and 3.10% of the total G57 H9N2 isolates, respectively (Supplementary Table S1). Notably, East China, Central China, and South China represent the largest poultry farming regions in China and also serve as crucial migratory pathways for wild birds (Olsen et al. 2006; Yang et al. 2020), which provides favorable conditions for the generation of novel reassortant viruses, such as H7N9, H10N8, and H3N8 (Chen et al. 2014, Lam et al. 2013; Yang et al. 2022). Vietnam, as the first country outside of China to detect the G57 H9N2 virus, has become a major source of the virus’s spread to neighboring countries, emphasizing the need for international cooperation in monitoring and controlling the cross-border spread of AIVs.

In addition, we found that the interspecies transmission pattern of G57 genotype H9N2 AIVs has become increasingly intricate. The cross-species transmission analysis shows that chickens are the primary hosts responsible for the spread of G57 AIVs, followed by domestic ducks. Strong BF support was found for transmission from chickens to other hosts, emphasizing their key role in virus spread. Additionally, significant BF support for transmission from ducks to chickens suggests a bidirectional transmission pattern between these poultry species, which could further facilitate the generation of new reassortants within the H9N2 lineage. The effect of the bidirectional transmission effect has been observed in the evolution and global spread of H5N1 within GS/96 lineage (Xie et al. 2023, Lin et al. 2024). There is another concern that both chickens and minor poultry contribute to transmit BJ/94 lineage H9N2 viruses to wild birds. Although not commonly found in wild bird populations, BJ/94 lineage H9N2 viruses have been isolated from wild birds outside China. Thus, close monitoring of the BJ/94 H9N2 virus in wild birds is essential to mitigate the risk of further international spread.

The comparison between earlier non-G57 H9N2 viruses and the G57 H9N2 viruses reveals a critical shift around 2012, marking the emergence of a novel reassortment and evolution pattern. Before 2012, the BJ/94 lineage of H9N2 virus was extensively involved in genetic reassortment with various subtypes of AIVs, primarily through exchanging partial internal genes. However, since 2013, this lineage has almost exclusively engaged in reassortment by exchanging all six internal genes, generating multiple novel strains capable of infecting both humans and animals, such as H7N9 (Pu et al. 2015). Additionally, before 2012, only H5 and H9 subtypes had caused infections in humans in China (Guan et al. 1999, Peiris et al. 1999), but after 2012, several new reassortants capable of infecting humans have emerged, including H3N8, H7N9, H10N8, and H10N3 (Gao et al. 2013; Qi et al. 2014; 2022; Yang et al. 2022). Moreover, these newly reassorting viruses will continue to pose an ongoing public health threat as they establish themselves as stable endemic agents within the chicken population (Bi et al. 2024, Kang et al. 2024).

The observed increase in zoonotic infections is primarily attributed to the widespread prevalence of the G57 genotype, which facilitates extensive exposure of the virus to a larger population, thereby significantly augmenting the likelihood of human infection. However, the actual number of H9N2 infections in humans is likely underestimated due to the typically mild symptoms associated with these infections (He et al. 2020; Qi et al. 2021, Song and Qin 2020). Additionally, adaptive mutations in G57 likely enhance the virus’s ability to spread among chickens and mammals, further increasing the public health risk. For example, various amino acid changes in HA protein after 2012 are experimentally demonstrated to impact antigenicity and host adaptability. Mutations at positions of 145, 149, 164, 220, and 239 of H9N2 HA protein can change viral antigenicity, which may contribute to the immune escape of H9N2 (Liu et al. 2023; Zhang et al. 2023b; 2023a). The R164Q mutation also increased viral replication in avian and mammalian cells (Zhang et al. 2023c; 2023a). In addition to the HA protein, adaptive mutations in the internal genes of G57 (like PB2-I292V, PA-K356R, and M1-T37A) enhance its ability to infect chickens, mice, or human cells for H9N2 and its reassortants (Cheng et al. 2014, Czudai-Matwich et al. 2014; Gao et al. 2019, Wang et al. 2022, Xiao et al. 2016; Xu et al. 2016).

In all, the results of this study deepened our understanding of the evolution and spread of BJ/94 lineage H9N2 AIVs, allowing us to identify key hosts and locations that may influence the increased viral risk. These findings highlight key areas that can inform strategies to reduce the impact of H9N2 viruses and protect public health.

## Supplementary Material

veae106_Supp

## Data Availability

Data are available in the main text and the supplementary materials.
