# Supplementary material for: Origin, spread, and interspecies transmission of a dominant genotype of BJ/94 lineage H9N2 avian influenza viruses with increased threat
Source: Virus Evol. 2024 Dec 9;10(1):veae106. doi: 10.1093/ve/veae106 (PMC11673197; doi:10.1093/ve/veae106)
Supplement: veae106_Supp [file veae106_supp.zip › suppl_data/Supplementary Figure 1-8.pdf]

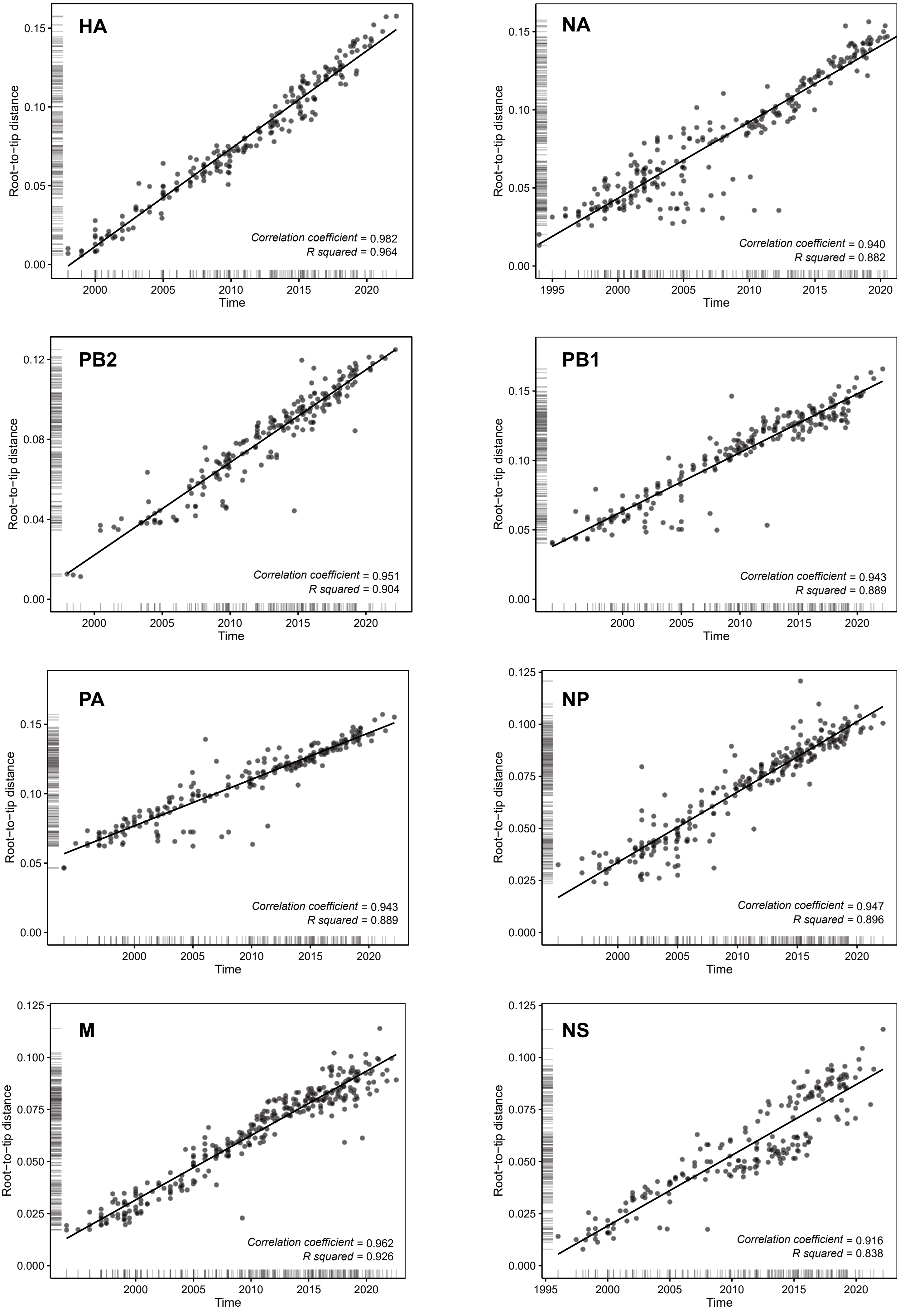
HA
Correlation coefficient = 0.982
R squared = 0.964
NA
Correlation coefficient = 0.940
R squared = 0.882
PB2
Correlation coefficient = 0.951
R squared = 0.904
PB1
Correlation coefficient = 0.943
R squared = 0.889
PA
Correlation coefficient = 0.943
R squared = 0.889
NP
Correlation coefficient = 0.947
R squared = 0.896
M
Correlation coefficient = 0.962
R squared = 0.926
NS
Correlation coefficient = 0.916
R squared = 0.838
Root-to-tip distance
Time

Supplementary Figure 1. Temporal signal detection in subsampled origin datasets. Strong temporal signal tested in TempEst of subsampled origin datasets.

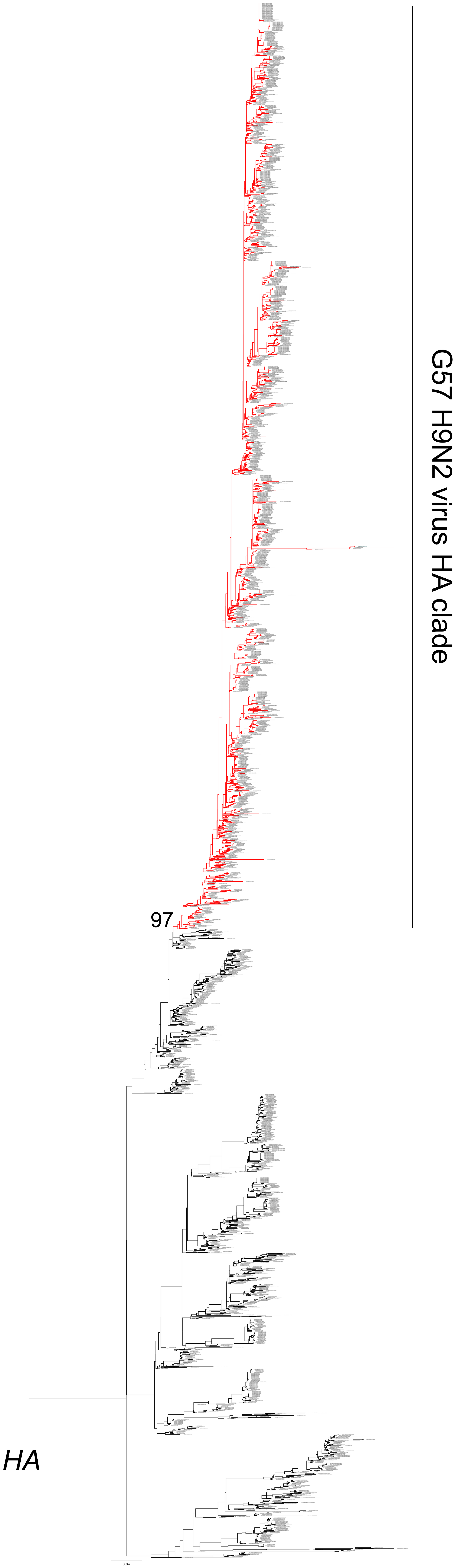

G57 H9N2 virus HA clade
97
HA
0.04

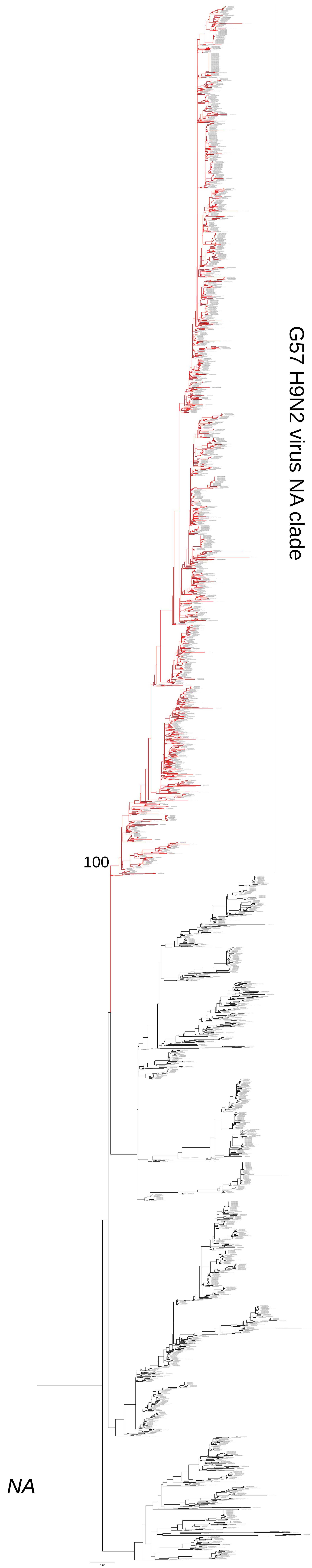
G57 H9N2 virus NA clade
100
NA

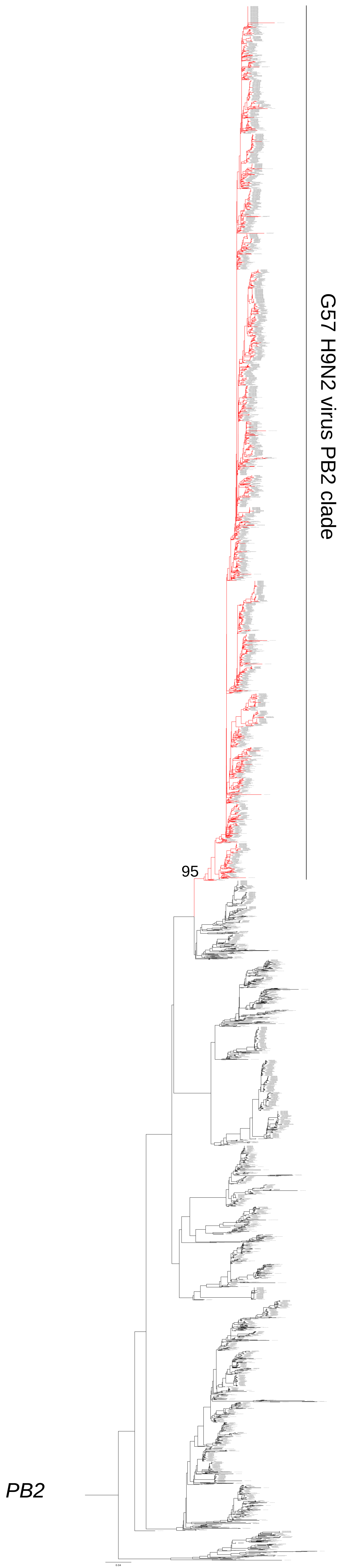

G57 H9N2 virus PB2 clade
95
PB2

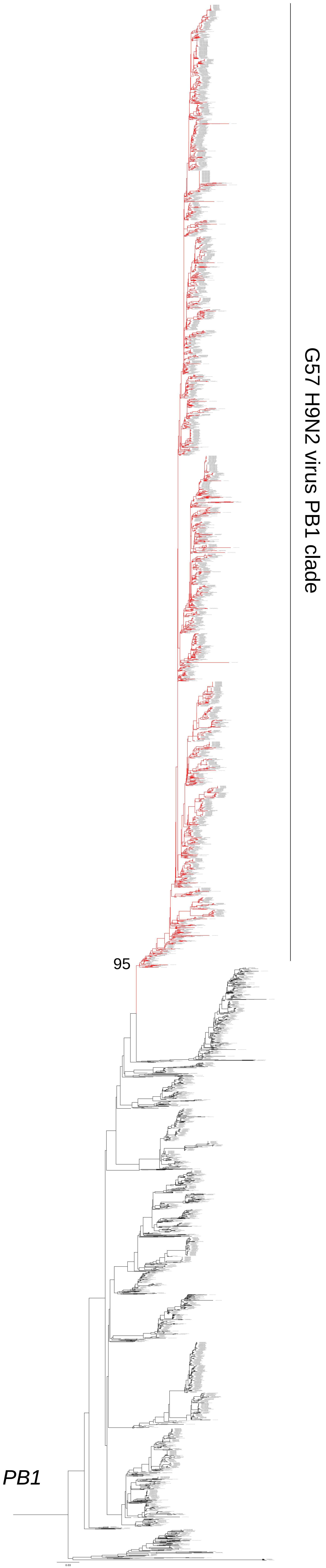
G57 H9N2 virus PB1 clade
95
PB1

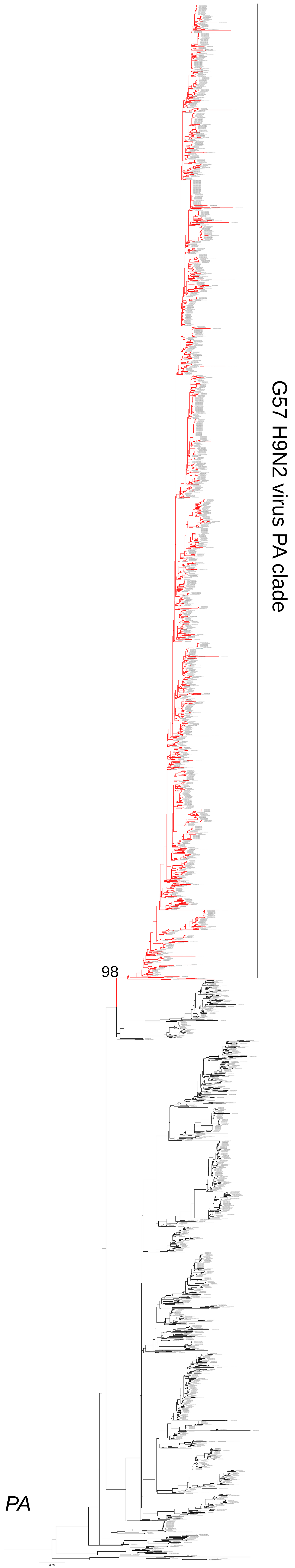
G57 H9N2 virus PA clade
98
PA

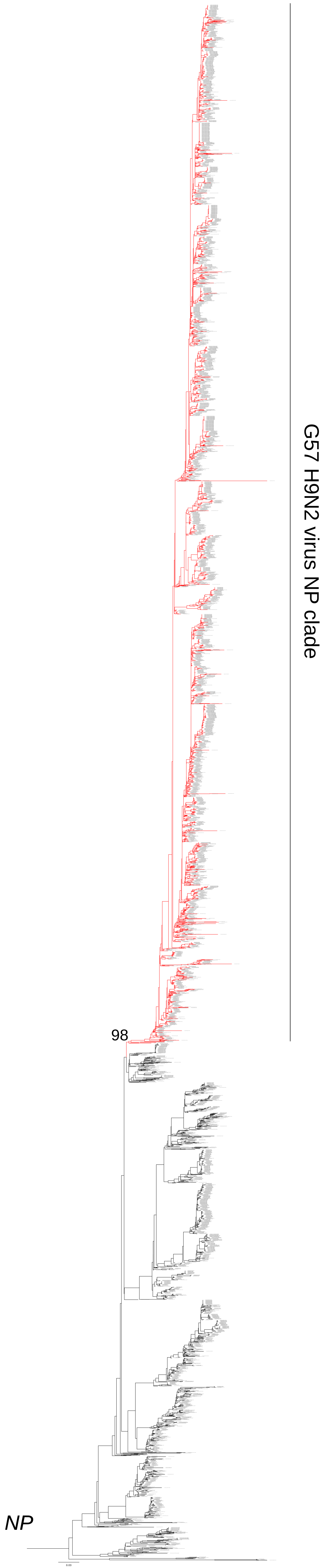
98
G57 H9N2 virus NP clade
NP

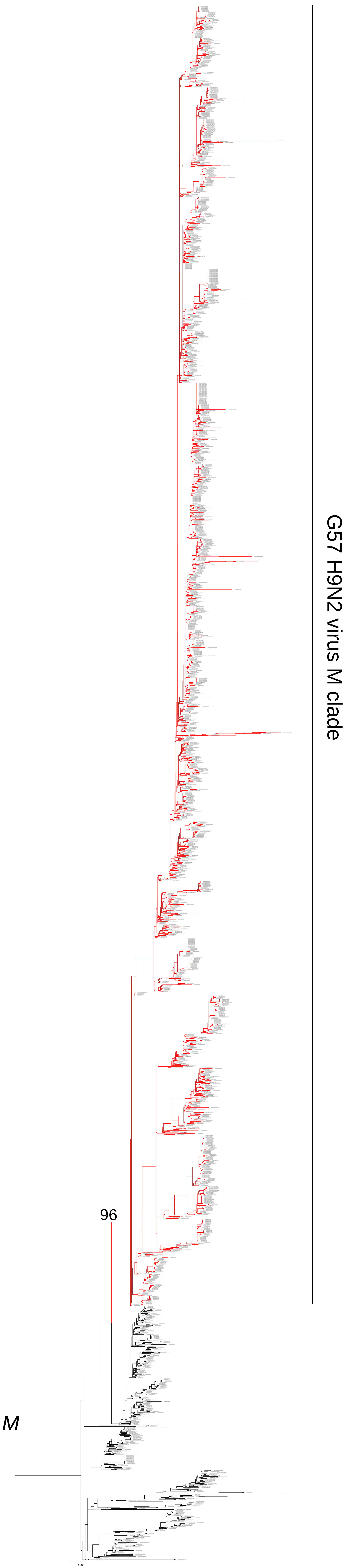

G57 H9N2 virus M clade
96
M
0.02

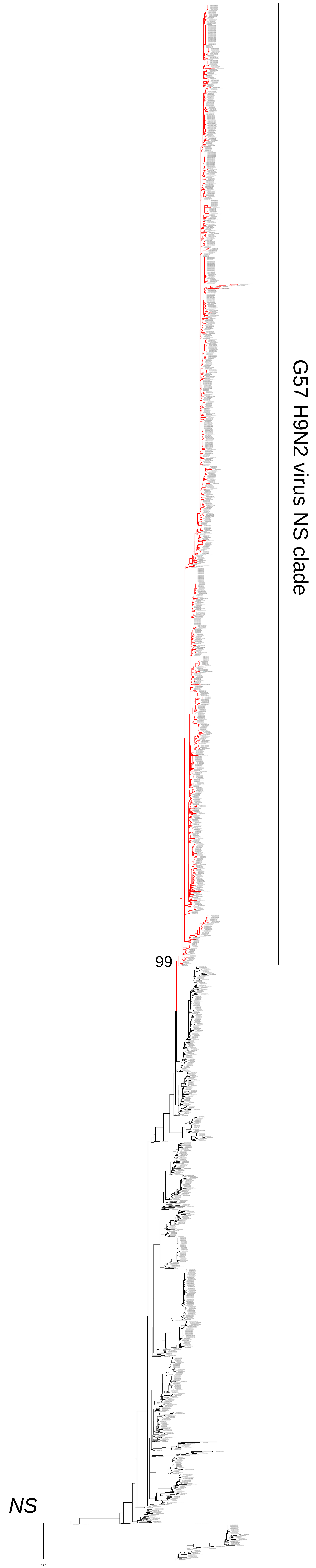
G57 H9N2 virus NS clade
99
NS

Supplementary Figure 2. Phylogenetic analysis of global H9N2 virus eight gene sequences. Phylogenetic tree of PB2, PB1, PA, HA, NP, NA, M and NS genes. The evolutionary tree includes the genome sequence of global H9N2 viruses (downloaded on March 30, 2023), where the branch to which the G57 H9N2 virus belongs is labeled in red and its bootstrap value is shown.

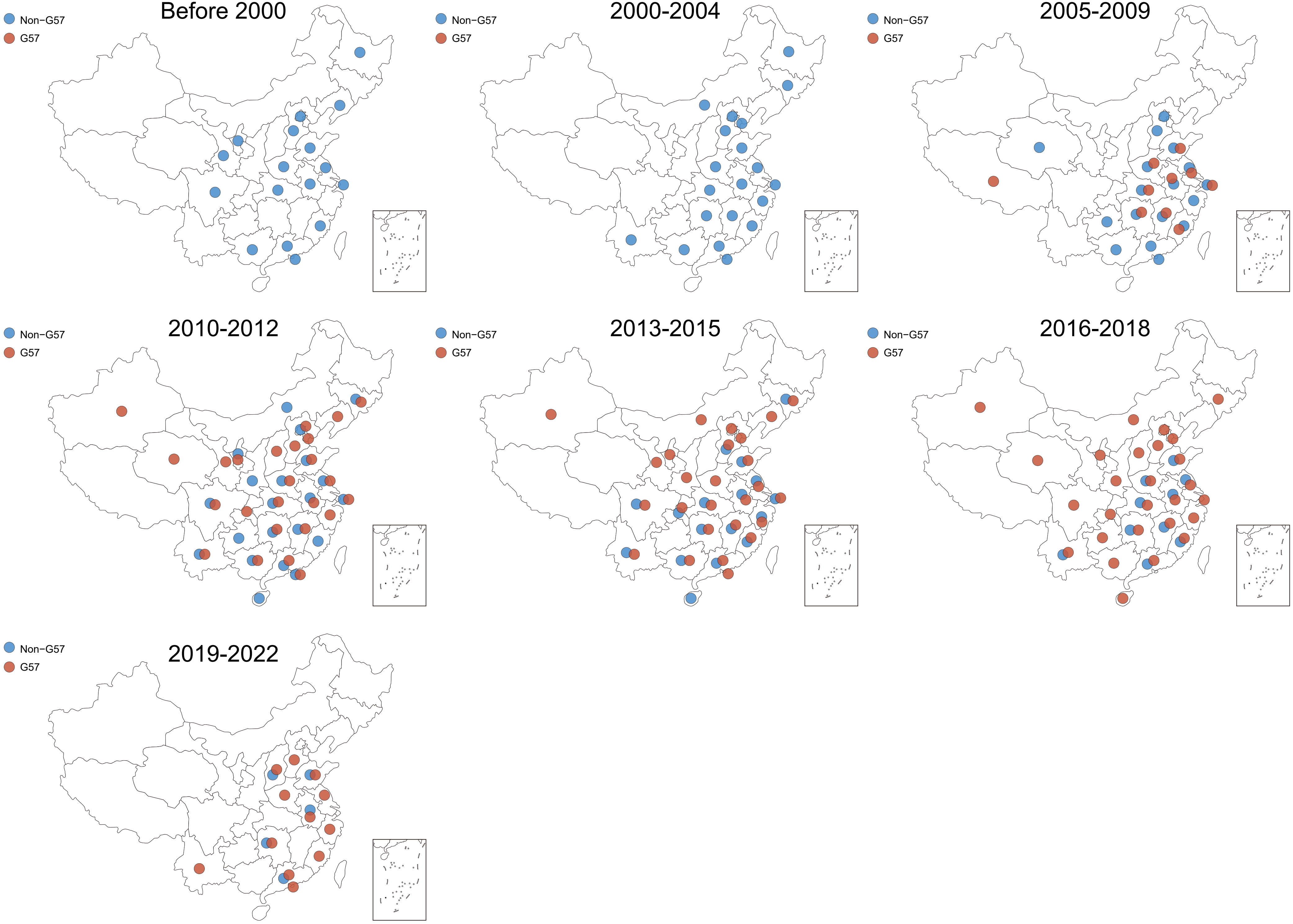
Before 2000
Non−G57
G57
2000-2004
Non−G57
G57
2005-2009
Non−G57
G57
2010-2012
Non−G57
G57
2013-2015
Non−G57
G57
2016-2018
Non−G57
G57
2019-2022
Non−G57
G57

Supplementary Figure 3. Epidemic range of G57 and non-G57 H9N2 viruses in China. The maps show the prevalent provinces of G57 and non-G57 H9N2 viruses in China at different periods of time.

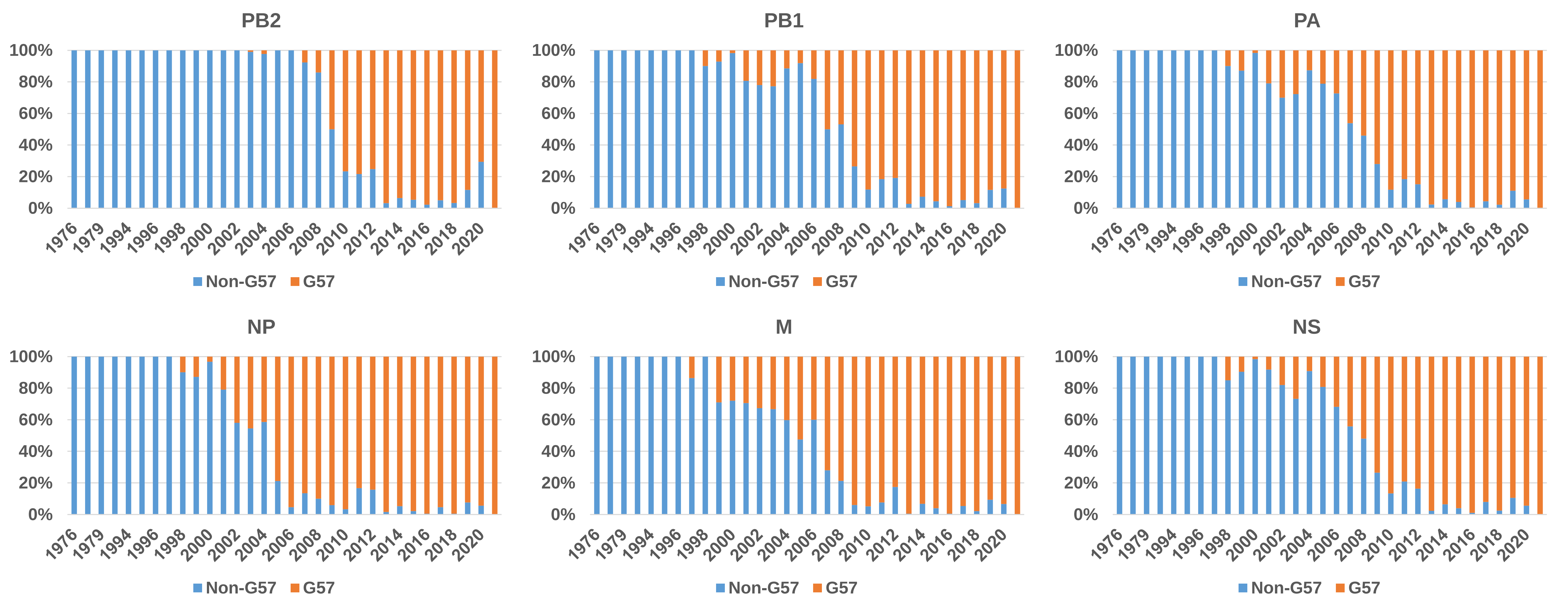
PB2
PB1
PA
NP
M
NS
100%
80%
60%
40%
20%
0%
1976
1979
1994
1996
1998
2000
2002
2004
2006
2008
2010
2012
2014
2016
2018
2020
Non-G57
G57

Supplementary Figure 4. Percentage distribution of non-G57 and G57 clade of H9N2 internal segments over time in China.

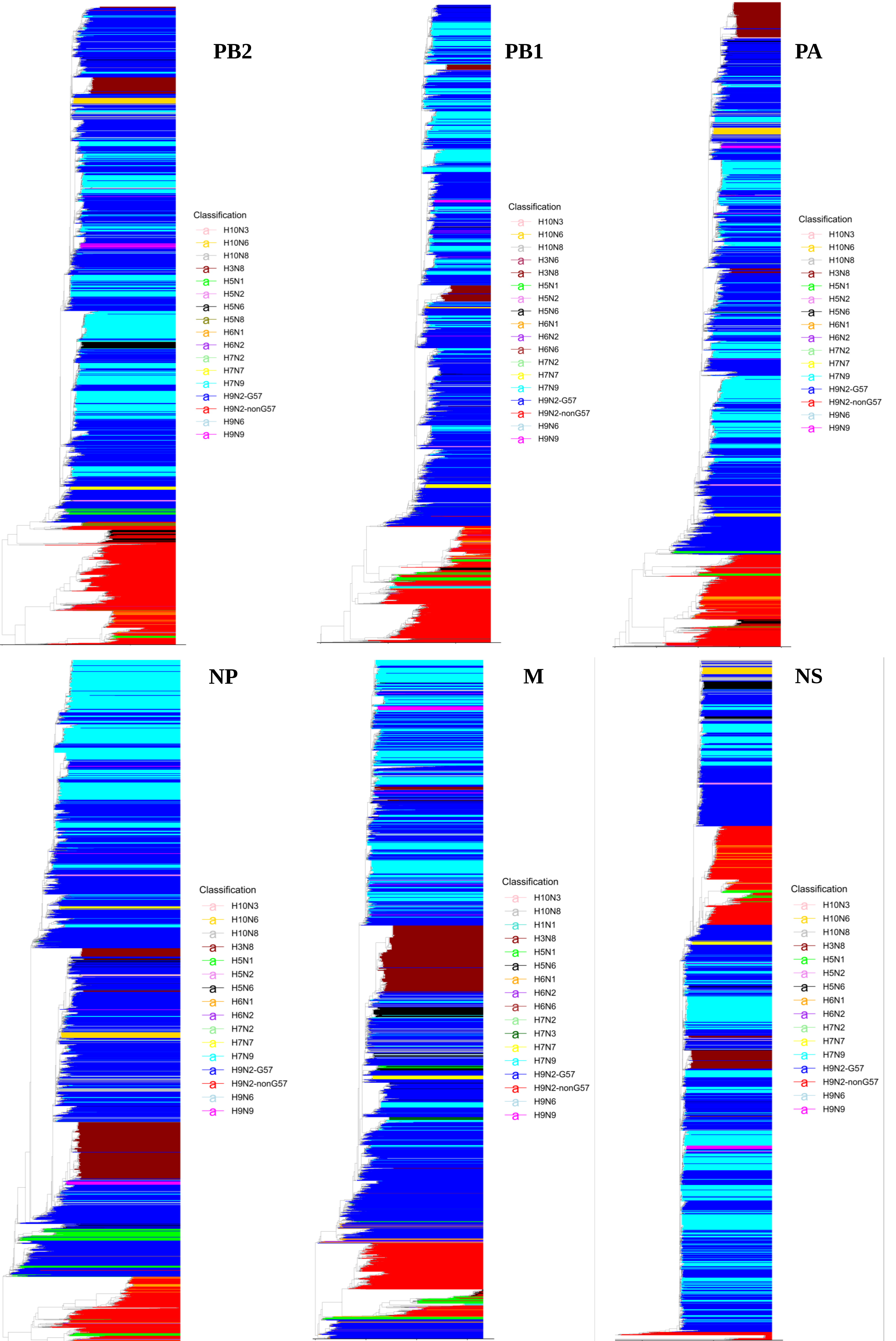
PB2
Classification
H10N3
H10N6
H10N8
H3N8
H5N1
H5N2
H5N6
H5N8
H6N1
H6N2
H7N2
H7N7
H7N9
H9N2-G57
H9N2-nonG57
H9N6
H9N9
PB1
Classification
H10N3
H10N6
H10N8
H3N6
H3N8
H5N1
H5N2
H5N6
H6N1
H6N2
H6N6
H7N2
H7N7
H7N9
H9N2-G57
H9N2-nonG57
H9N6
H9N9
PA
Classification
H10N3
H10N6
H10N8
H3N8
H5N1
H5N2
H5N6
H6N1
H6N2
H7N2
H7N7
H7N9
H9N2-G57
H9N2-nonG57
H9N6
H9N9
NP
Classification
H10N3
H10N6
H10N8
H3N8
H5N1
H5N2
H5N6
H6N1
H6N2
H7N2
H7N7
H7N9
H9N2-G57
H9N2-nonG57
H9N6
H9N9
M
Classification
H10N3
H10N8
H1N1
H3N8
H5N1
H5N6
H6N1
H6N2
H6N6
H7N2
H7N3
H7N7
H7N9
H9N2-G57
H9N2-nonG57
H9N6
H9N9
NS
Classification
H10N3
H10N6
H10N8
H3N8
H5N1
H5N2
H5N6
H6N1
H6N2
H7N2
H7N7
H7N9
H9N2-G57
H9N2-nonG57
H9N6
H9N9

Supplementary Figure 5. Maximum-likelihood tree of high homology subtypes with H9N2 internal segments in China. H9N2-G57 label indicates that the internal segment of H9N2 viruses belongs to the G57 clade. AIV highly homologous to each internal segment of H9N2 are marked by different colors on ML tree.

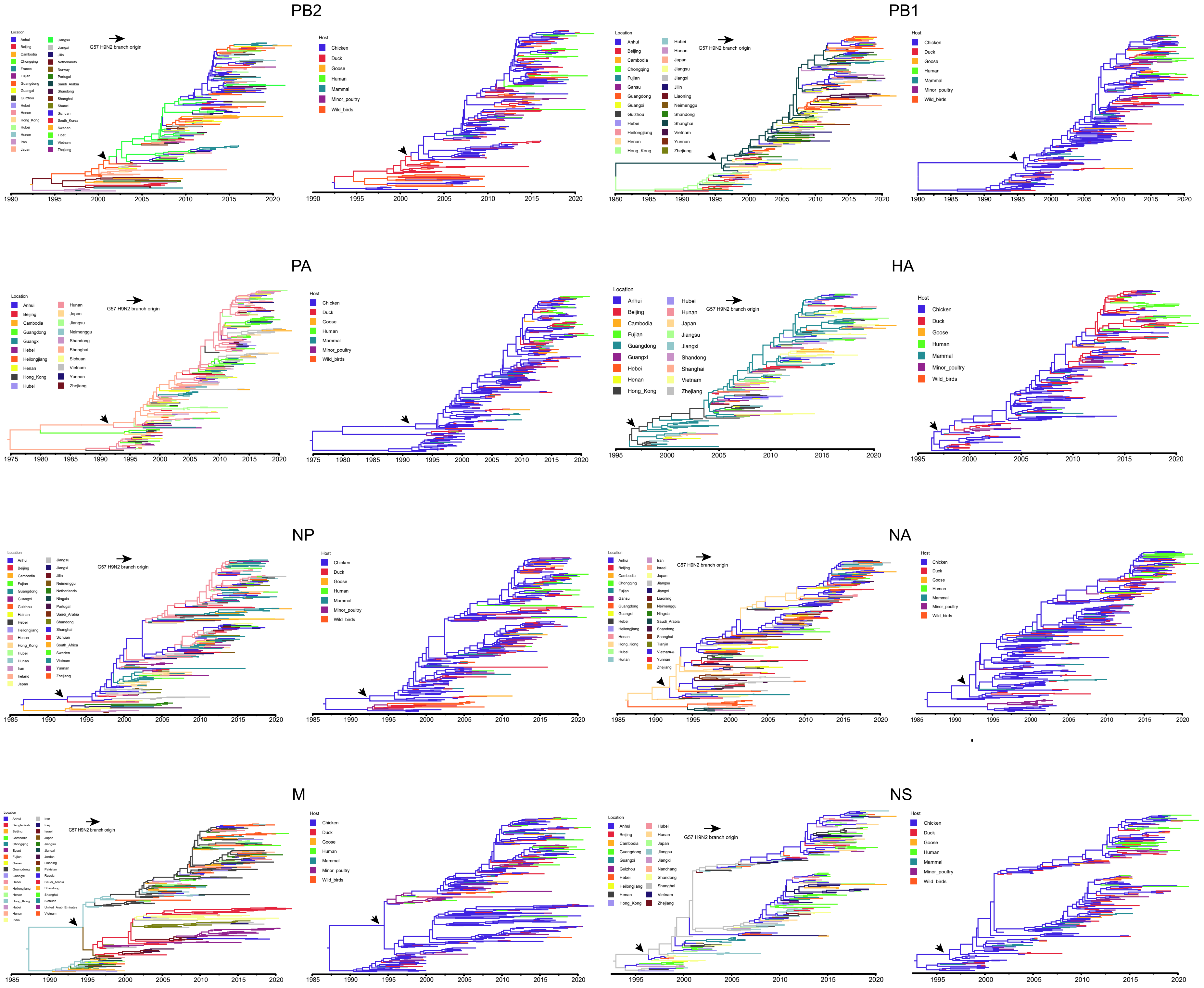
PB2
PB1
PA
HA
NP
NA
M
NS
G57 H9N2 branch origin
Location
Host
Chicken
Duck
Goose
Human
Mammal
Minor_poultry
Wild_birds
Anhui
Beijing
Cambodia
Fujian
Guangdong
Guangxi
Hebei
Henan
Hong_Kong
Hubei
Hunan
Japan
Jiangsu
Jiangxi
Shandong
Shanghai
Vietnam
Zhejiang
1990
1995
2000
2005
2010
2015
2020

Supplementary Figure 6. MCC tree of H9N2 virus for eight gene origin datasets. Maximum clade credibility trees of PB2, PB1, PA, HA, NP, NA, M, and NS genes subsampled origin datasets with branches colored by geographic regions or hosts.

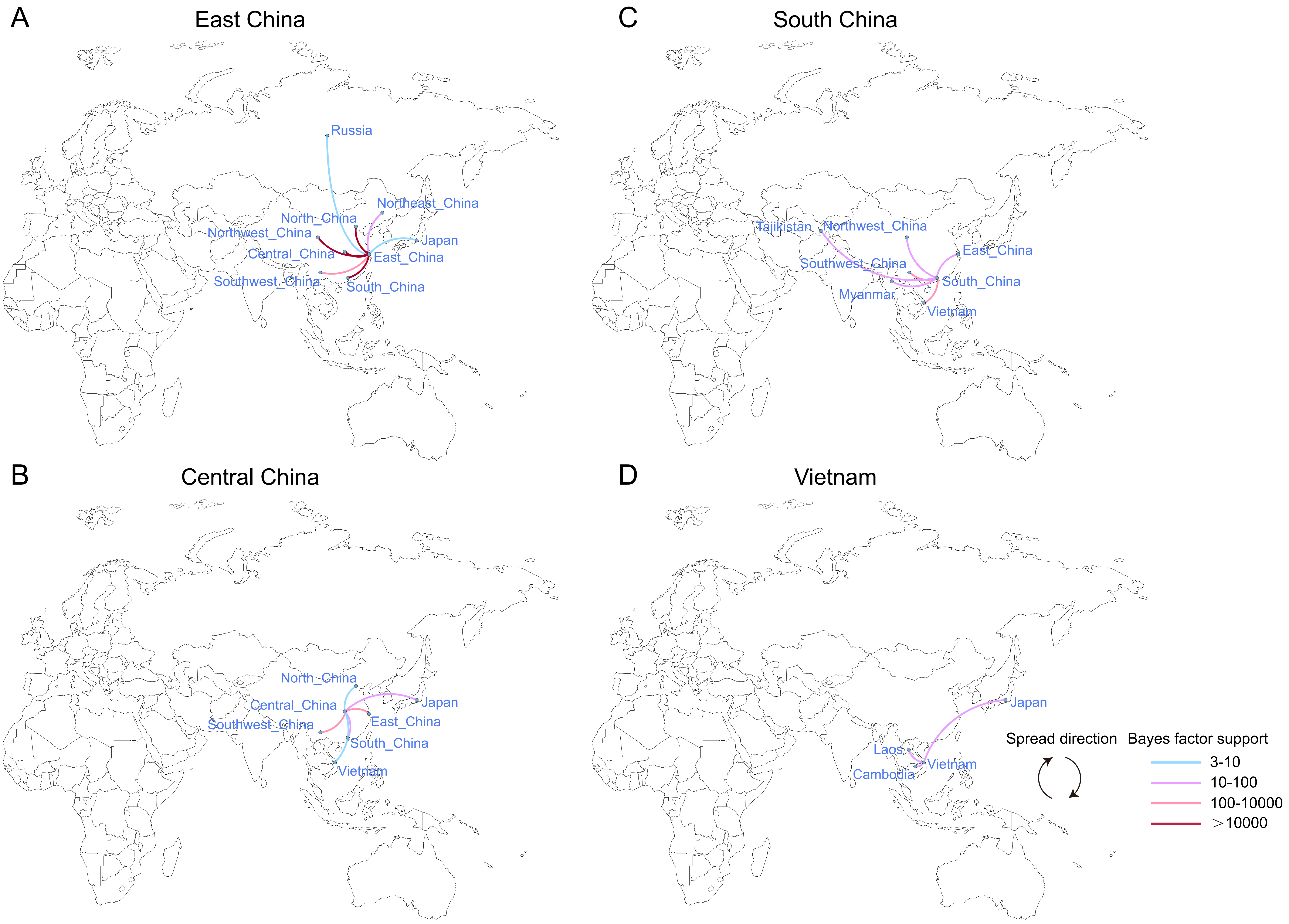

A
East China
Russia
Northeast_China
North_China
Northwest_China
Japan
Central_China
East_China
Southwest_China
South_China
B
Central China
North_China
Japan
Central_China
East_China
Southwest_China
South_China
Vietnam
C
South China
Tajikistan
Northwest_China
East_China
Southwest_China
South_China
Myanmar
Vietnam
D
Vietnam
Japan
Laos
Vietnam
Cambodia
Spread direction
Bayes factor support
3-10
10-100
100-10000
>10000

Supplementary Figure 7. Geographic transition of major sink regions. Transitions and Bayes factors of transmission from East China, Central China, South China and Vietnam to other region taxa.

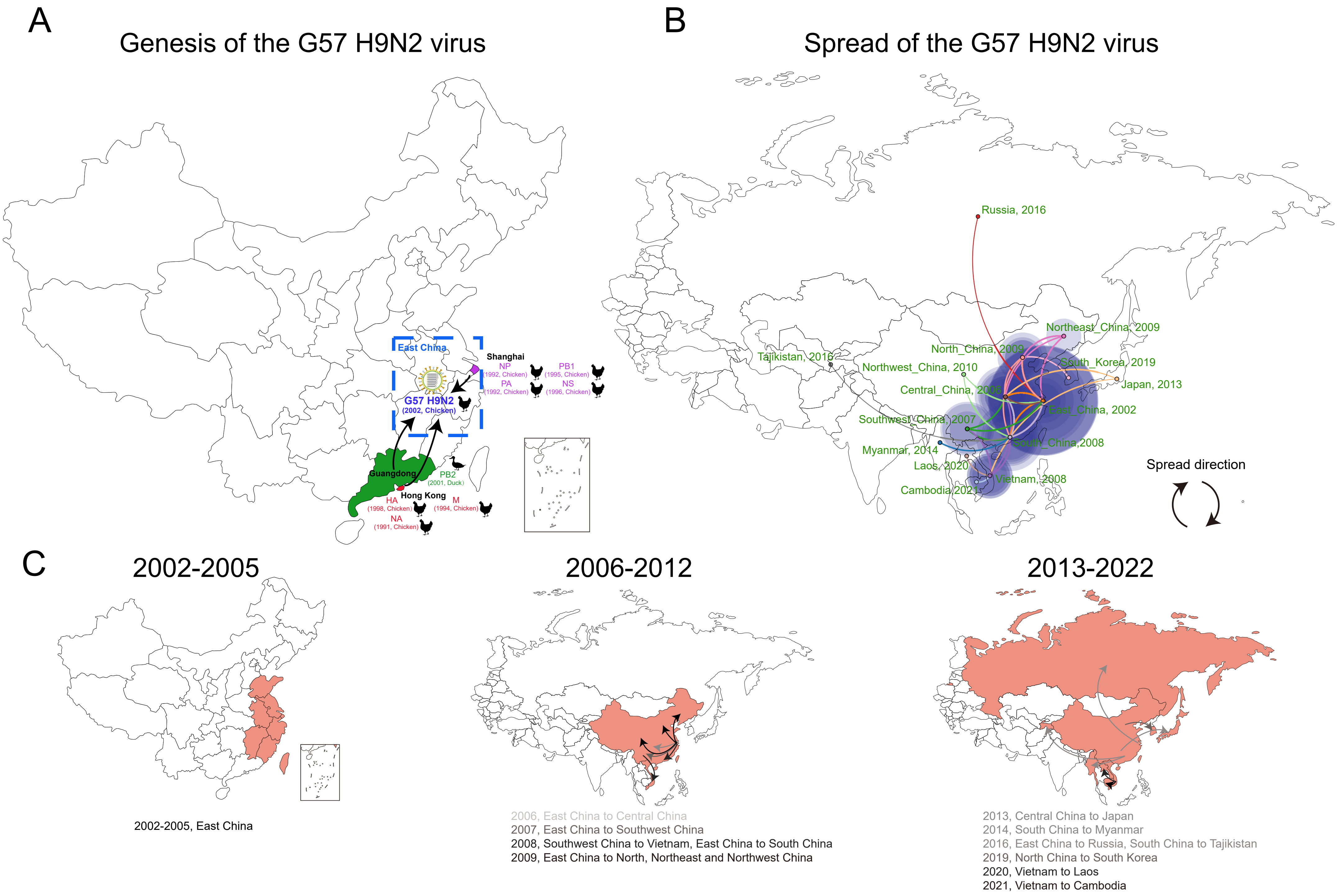
A
Genesis of the G57 H9N2 virus
East China
Shanghai
NP
(1992, Chicken)
PB1
(1995, Chicken)
PA
(1992, Chicken)
NS
(1996, Chicken)
G57 H9N2
(2002, Chicken)
Guangdong
PB2
(2001, Duck)
Hong Kong
HA
(1998, Chicken)
M
(1994, Chicken)
NA
(1991, Chicken)
B
Spread of the G57 H9N2 virus
Russia, 2016
Northeast_China, 2009
North_China, 2009
Tajikistan, 2016
Northwest_China, 2010
South_Korea, 2019
Japan, 2013
Central_China, 2006
East_China, 2002
Southwest_China, 2007
South_China,2008
Myanmar, 2014
Laos, 2020
Vietnam, 2008
Cambodia,2021
Spread direction
C
2002-2005
2006-2012
2013-2022
2002-2005, East China
2006, East China to Central China
2007, East China to Southwest China
2008, Southwest China to Vietnam, East China to South China
2009, East China to North, Northeast and Northwest China
2013, Central China to Japan
2014, South China to Myanmar
2016, East China to Russia, South China to Tajikistan
2019, North China to South Korea
2020, Vietnam to Laos
2021, Vietnam to Cambodia

Supplementary Figure 8. Summary of the G57 H9N2 virus origin, and transmission pathways. (A) Schematic representation of the origin of the G57 H9N2 virus. (B) Inferred transmission pathway of the G57 H9N2 virus. The color of the line is consistent with the dispersed region, and the purple shaded circle indicates the intensity of transmission in the region, with a larger radius contributing to greater virus transmission. The numbers represent the inferred time of introduction of the virus in the region (in years). (C-E) Transmission of the G57 H9N2 virus in various time periods.
