# Supplementary material for: Origin, spread, and interspecies transmission of a dominant genotype of BJ/94 lineage H9N2 avian influenza viruses with increased threat
Source: Virus Evol. 2024 Dec 9;10(1):veae106. doi: 10.1093/ve/veae106 (PMC11673197; doi:10.1093/ve/veae106)
Supplement: veae106_Supp [file veae106_supp.zip › suppl_data/Supplementary Table 2-6.docx]

**Supplementary Table 2. Classification of Chinese provinces in this study**

| **Region** | **Province** |
| --- | --- |
| Northeast China | Heilongjiang, Jilin, Liaoning |
| East China | Shandong, Jiangsu, Shanghai, Zhejiang, Anhui, Jiangxi, Fujian, Taiwan |
| North China | Hebei, Beijing, Tianjin, Shanxi, Neimenggu |
| Central China | Henan, Hubei, Hunan |
| South China | Guangdong, Guangxi, Hainan, Hong Kong, Macau |
| Southwest China | Sichuan, Yunnan, Guizhou, Chongqing, Xizang |
| Northwest China | Xinjiang, Ningxia, Qinghai, Shaanxi, Gansu |

**Supplementary Table 3. Temporal, host and spatial distribution of the G57 H9N2 virus**

| **Year** | **Counts** | **Host** | **Location** |
| --- | --- | --- | --- |
| 2007 | 2 | Chicken | Jiangxi, Zhejiang |
| 2008 | 4 | Chicken, Mammal | Anhui, Jiangsu, Shandong |
| 2009 | 29 | Chicken, Duck, Mammal, Minor_poultry, Wild_birds | Anhui, Henan, Hubei, Hunan, Jiangsu, Shandong, Shanghai, Tibet, Vietnam, Zhejiang |
| 2010 | 38 | Chicken, Mammal | Anhui, Beijing, China, Guangxi, Hebei, Hong_Kong, Hunan, Jiangsu, Shandong, Shanghai, Zhejiang |
| 2011 | 67 | Chicken, Duck, Minor_poultry | Anhui, Chongqing, Hebei, Henan, Hong_Kong, Hubei, Hunan, Jiangsu, Jiangxi, Ningxia, Qinghai, Shandong, Shanghai, Sichuan, Yunnan, Zhejiang |
| 2012 | 99 | Chicken, Duck, Goose, Minor_poultry, Wild_birds | Anhui, Beijing, China, Gansu, Guangdong, Guangxi, Hebei, Henan, Hubei, Hunan, Jiangsu, Jiangxi, Jilin, Liaoning, Shandong, Shanghai, Shanxi, Tianjin, Vietnam, Xinjiang, Zhejiang |
| 2013 | 424 | Chicken, Duck, Goose, Human, Minor_poultry, Wild_birds | Anhui, Beijing, China, Chongqing, Fujian, Gansu, Guangdong, Guangxi, Hebei, Henan, Hong_Kong, Hubei, Hunan, Jiangsu, Jiangxi, Jilin, Liaoning, Shaanxi, Shandong, Shanghai, Sichuan, Tianjin, Vietnam, Zhejiang |
| 2014 | 243 | Chicken, Duck, Mammal, Minor_poultry | Anhui, China, Chongqing, Fujian, Gansu, Guangdong, Guangxi, Hebei, Henan, Hubei, Hunan, Jiangsu, Jiangxi, Jilin, Neimenggu, Ningxia, Shandong, Shanghai, Sichuan, Vietnam, Yunnan, Zhejiang |
| 2015 | 254 | Chicken, Duck, Goose, Human, Mammal, Minor_poultry, Wild_birds | Anhui, China, Fujian, Guangdong, Guangxi, Henan, Hubei, Hunan, Japan, Jiangsu, Jiangxi, Jilin, Myanmar, Shandong, Shanghai, Sichuan, Xinjiang, Yunnan, Zhejiang |
| 2016 | 180 | Chicken, Duck, Goose, Human, Mammal, Minor_poultry, Wild_birds | Anhui, Beijing, China, Fujian, Guangdong, Guizhou, Hebei, Henan, Hubei, Hunan, Japan, Jiangsu, Jiangxi, Ningxia, Shandong, Shanghai, Shanxi, Sichuan, Vietnam, Yunnan, Zhejiang |
| 2017 | 445 | Chicken, Duck, Human, Minor_poultry, Wild_birds | Anhui, Beijing, China, Fujian, Guangdong, Guizhou, Hainan, Henan, Hubei, Hunan, Japan, Jiangsu, Jiangxi, Jilin, Shandong, Shanghai, Shanxi, Tianjin, Vietnam, Xinjiang, Yunnan |
| 2018 | 473 | Chicken, Duck, Human, Mammal, Minor_poultry | Anhui, China, Chongqing, Fujian, Guangdong, Guangxi, Guizhou, Hainan, Henan, Hunan, Jiangsu, Jiangxi, Jilin, Neimenggu, Qinghai, Russia, Shaanxi, Shandong, Shanghai, Shanxi, Tajikistan, Vietnam, Yunnan, Zhejiang |
| 2019 | 139 | Chicken, Duck, Human, Mammal, Minor_poultry, Wild_birds | Anhui, China, Fujian, Guangdong, Hebei, Henan, Hong_Kong, Hubei, Jiangsu, Shandong, Shanxi, Vietnam, Yunnan, Zhejiang |
| 2020 | 24 | Chicken, Duck, Human, Wild_birds | China, Fujian, Guangdong, Hunan, Jiangsu, Laos, South_Korea, Vietnam |
| 2021 | 3 | Chicken, Human | China, Jiangsu |
| 2022 | 1 | Human | Cambodia |

*Note*: "Mammal" includes Mink, Swine, Fox, Raccoon dog and Cat; "Minor_poultry" includes Quail, Pigeon, Ostrich, Pheasant, etc. The first isolation of G57 H9N2 virus in a country other than China is indicated in blue.

**Supplementary Table 4.** **Inferred origin time, region and host of the G57 H9N2 virus**

| **Segment** | **Time to most recent common ancestor (TMRCA)** | **95% highest**  **posterior density (HPD)** | **Inferred most likely region of origin (probability)** | **Inferred most likely host of origin (probability)** |
| --- | --- | --- | --- | --- |
| PB2 | March 19, 2001 | August 1, 2000 to November 11, 2001 | Guangdong (95.24%) | Duck (90.18%) |
| PB1 | December 10, 1995 | March 2, 1995 to August 28, 1996 | Shanghai (95.27%) | Chicken (99.93%) |
| PA | April 17, 1992 | June 30, 1990 to November 19, 1993 | Shanghai (83.62%) | Chicken (97.03%) |
| HA | April 4, 1997 | October 29, 1996 to September 28, 1997 | Hong Kong (96.53%) | Chicken (93.92%) |
| NP | July 19, 1992 | June 30, 1990 to July 15, 1994 | Shanghai (80.46%) | Chicken (83.98%) |
| NA | December 22, 1991 | January 13, 1991 to December 20, 1992 | Hong Kong (72.82%) | Chicken (99.99%) |
| M | June 17, 1994 | September 16, 1993 to August 2, 1995 | Hong Kong (62.12%) | Chicken (68.70%) |
| NS | May 10, 1996 | September 10, 1995 to March 8, 1997 | Shanghai (98.92%) | Chicken (99.85%) |

**Supplementary Table 5. Geographical transitions and reward time of the G57 H9N2 virus.**

| **Region Transition** | | | | **Region Rewards** | | |
| --- | --- | --- | --- | --- | --- | --- |
| **Transition** | **Markov Jumps** | **Percentage** | **Bayes Factor** | **Region** | **Reward Time** | **Percentage** |
| East_China to North_China | 21.92 | 13.13% | 215349.80 | East_China | 358.73 | 55.63% |
| East_China to South_China | 24.81 | 14.86% | 215349.80 | Central_China | 94.09 | 14.59% |
| East_China to Central_China | 23.98 | 14.36% | 215349.80 | South_China | 83.72 | 12.98% |
| East_China to Northwest_China | 9.06 | 5.43% | 17933.63 | North_China | 29.41 | 4.56% |
| Central_China to East_China | 26.07 | 15.61% | 5508.84 | Vietnam | 27.47 | 4.26% |
| South_China to Southwest_China | 4.80 | 2.88% | 1063.52 | Southwest_China | 25.43 | 3.94% |
| South_China to Vietnam | 5.30 | 3.18% | 824.70 | Northeast_China | 7.44 | 1.15% |
| Central_China to Southwest_China | 5.50 | 3.29% | 211.98 | Northwest_China | 7.09 | 1.10% |
| North_China to Northeast_China | 5.02 | 3.01% | 180.21 | Japan | 5.84 | 0.91% |
| East_China to Southwest_China | 7.34 | 4.40% | 147.07 | Russia | 1.11 | 0.17% |
| Vietnam to Cambodia | 0.91 | 0.55% | 91.81 | Tajikistan | 1.10 | 0.17% |
| Vietnam to Japan | 0.98 | 0.58% | 72.41 | Cambodia | 1.03 | 0.16% |
| Vietnam to Laos | 0.91 | 0.55% | 63.82 | South_Korea | 1.02 | 0.16% |
| South_China to East_China | 6.31 | 3.78% | 60.56 | Myanmar | 0.93 | 0.14% |
| Central_China to South_China | 6.20 | 3.71% | 45.02 | Laos | 0.51 | 0.08% |
| Central_China to Japan | 2.24 | 1.34% | 37.48 |  |  |  |
| North_China to South_Korea | 0.73 | 0.44% | 33.52 |  |  |  |
| East_China to Northeast_China | 3.31 | 1.98% | 32.55 |  |  |  |
| South_China to Myanmar | 0.73 | 0.44% | 29.56 |  |  |  |
| Southwest_China to Vietnam | 2.07 | 1.24% | 28.53 |  |  |  |
| South_China to Tajikistan | 0.62 | 0.37% | 16.35 |  |  |  |
| South_China to Northwest_China | 1.17 | 0.70% | 12.59 |  |  |  |
| East_China to Russia | 0.32 | 0.19% | 7.51 |  |  |  |
| Central_China to North_China | 0.77 | 0.46% | 4.76 |  |  |  |
| East_China to Japan | 0.73 | 0.44% | 4.64 |  |  |  |
| Central_China to Vietnam | 0.70 | 0.42% | 4.30 |  |  |  |
| Northwest_China to Vietnam | 0.21 | 0.12% | 3.55 |  |  |  |

*Note:* Only transitions with BF >=3 is shown.

**Supplementary Table 6. Host transitions and reward time of the G57 H9N2 virus.**

| **Host Transition** | | | | **Host Reward** | | |
| --- | --- | --- | --- | --- | --- | --- |
| **Transition** | **Markov Jumps** | **Percentage** | **Bayes Factor** | **Host** | **Reward Time** | **Percentage** |
| Chicken to Duck | 40.07 | 40.05% | 47475.67 | Chicken | 564.37 | 86.83% |
| Chicken to Human | 16.68 | 14.83% | 47475.67 | Duck | 35.28 | 5.43% |
| Chicken to Minor_poultry | 24.74 | 21.98% | 47475.67 | Human | 20.50 | 3.15% |
| Chicken to Wild_birds | 11.65 | 10.36% | 47475.67 | Minor_poultry | 16.51 | 2.54% |
| Chicken to Goose | 3.98 | 3.54% | 4742.82 | Wild_birds | 7.80 | 1.20% |
| Chicken to Mammal | 6.06 | 5.39% | 963.72 | Mammal | 4.58 | 0.70% |
| Duck to Mammal | 0.83 | 0.93% | 10.23 | Goose | 1.66 | 0.26% |
| Minor_poultry to Wild_birds | 0.40 | 0.45% | 4.79 |  |  |  |
| Duck to Chicken | 1.61 | 1.43% | 4.29 |  |  |  |

*Note:* Only transitions with BF >=3 is shown.
